# Cutaneous Fungal Infections Caused by Dermatophytes and Non-Dermatophytes: An Updated Comprehensive Review of Epidemiology, Clinical Presentations, and Diagnostic Testing

**DOI:** 10.3390/jof9060669

**Published:** 2023-06-14

**Authors:** Pattriya Chanyachailert, Charussri Leeyaphan, Sumanas Bunyaratavej

**Affiliations:** Department of Dermatology, Faculty of Medicine Siriraj Hospital, Mahidol University, 2 Wanglang Road, Bangkok Noi, Bangkok 10700, Thailand; pattriya.cht@gmail.com (P.C.); charussrilee@gmail.com (C.L.)

**Keywords:** clinical, cutaneous fungal infection, dermatophyte, diagnosis, epidemiology, microsporum, non-dermatophyte, onychomycosis, tinea, trichophyton

## Abstract

Cutaneous fungal infection of the skin and nails poses a significant global public health challenge. Dermatophyte infection, mainly caused by *Trichophyton* spp., is the primary pathogenic agent responsible for skin, hair, and nail infections worldwide. The epidemiology of these infections varies depending on the geographic location and specific population. However, epidemiological pattern changes have occurred over the past decade. The widespread availability of antimicrobials has led to an increased risk of promoting resistant strains through inappropriate treatment. The escalating prevalence of resistant *Trichophyton* spp. infections in the past decade has raised serious healthcare concerns on a global scale. Non-dermatophyte infections, on the other hand, present even greater challenges in terms of treatment due to the high failure rate of antifungal therapy. These organisms primarily target the nails, feet, and hands. The diagnosis of cutaneous fungal infections relies on clinical presentation, laboratory investigations, and other ancillary tools available in an outpatient care setting. This review aims to present an updated and comprehensive analysis of the epidemiology, clinical manifestations, and diagnostic testing methods for cutaneous fungal infections caused by dermatophytes and non-dermatophytes. An accurate diagnosis is crucial for effective management and minimizing the risk of antifungal resistance.

## 1. Introduction

Cutaneous fungal infections, caused by dermatophyte and non-dermatophyte fungi, pose significant challenges to global public health. Dermatophyte infections primarily affect the skin, hair, and nails. In recent years, there has been a concerning rise in the prevalence of antifungal-resistant species within certain ethnic groups, further complicating the management of these infections. One example is the emergence of *Trichophyton indotineae* in the Indian population. Non-dermatophyte infections require specific diagnostic criteria to differentiate them from contamination. These organisms can infect the nails and skin on the hands and feet, presenting as potential pathogens. The occurrence of these two major pathogenic fungal infections is not uncommon.

Clinical manifestations can provide valuable guidance in identifying the specific fungal pathogen and initiating management. This is particularly relevant in regions where conventional techniques for fungal identification remain widely used, such as in middle to low-income countries.

This review aims to provide an updated literature analysis focusing on the epidemiology, clinical manifestations, and diagnostic testing of dermatophyte and non-dermatophyte cutaneous fungal infections.

## 2. Epidemiology

### 2.1. Dermatophyte Infections

Cutaneous fungal infections have been reported worldwide, affecting an estimated 20–25% of the global population [1]. In geographic regions with high prevalence, the incidence of dermatophytosis can reach as high as 40–60% [2,3]. Various factors, such as age, sex, climate, urban environment, socioeconomic level, and cultural habits can contribute to the occurrence of dermatophytosis.

In 1934, dermatophytes were classified by Chester Emmons into three genera based on spore morphology and accessory organs: *Trichophyton*, *Microsporum*, and *Epidermophyton* [4]. However, with advancements in phylogenetic analysis, a total of nine genera have now been identified: *Trichophyton*, *Epidermophyton*, *Nannizia*, *Paraphyton*, *Lopophyton*, *Microsporum*, *Arthroderma*, *Ctenomyces*, and *Guarromyces* [5]. Nevertheless, the primary pathogens that predominantly infect humans belong to the four genera of *Trichophyton*, *Microsporum*, *Epidermophyton*, and *Nannizzia*. Additionally, these organisms can be further classified based on their primary habitat into anthropophilic, zoophilic, and geophilic species (see Table 1).

In the 20th century, *Microsporum audouinii* and *Trichophyton schoenleinii* were the primary pathogenic organisms responsible for tinea capitis in the British Isles, Northern and Western Europe, and the Americas before 1950 [7]. During that period, *Trichophyton mentagrophytes* were the leading cause of tinea pedis and tinea corporis. By the late 20th century, *T. rubrum* had emerged as the predominant global agent, with a prevalence of over 40–70% in Central and North European countries, followed by *T. mentagrophytes* [8,9]. This trend was similar in the United States of America and Southeast Asia [10,11]. Southern Europe, on the other hand, had *Microsporum canis* and *Trichophyton verrucosum* as the most commonly isolated zoophilic dermatophytes [8]. In Western Asia, the most frequently isolated organism was *Epidermophyton floccosum* [12], while *Trichophyton violaceum* was the chief causative agent in Africa [13].

Moving into the 21st century, anthropophilic dermatophytes such as *E. floccosum*, *M. audouinii*, and *T. schoenleinii* saw a decline in prevalence in European countries, being replaced by various *Trichophyton* species [14]. *T. rubrum* emerged as the predominant organism worldwide, affecting Europe, South America, Asia, and Africa. The incidence of *T. rubrum* infection has significantly increased since the 20th century, predominantly affecting individuals between the ages of 20 and 60, with a tendency to infect older patients in more recent years [15]. *T. rubrum* is responsible for a wide range of fungal infections, including tinea pedis, onychomycosis, tinea cruris, tinea corporis, and tinea manuum [3,15,16,17,18,19,20,21,22]. The most commonly affected areas are the feet and toenails. Several risk factors contribute to *T. rubrum* infection, including the use of occlusive footwear and hot, humid weather conditions, which are particularly conducive to the spread of the infection during the summer season [15]. Other causative organisms vary depending on the site of infection and regional factors, as outlined in Table 2 [3,16,17,18,19,20,21,22].

### 2.2. Non-Dermatophyte Infections

In addition to dermatophyte infections, non-dermatophytes have long been acknowledged as saprophytic organisms prevalent in soil and the environment. They are renowned for their ability to flourish in fungal culture media. However, recent reports have identified them as causative pathogens in cases of onychomycosis, tinea pedis, and tinea manuum. To differentiate these organisms as genuine pathogens rather than mere contaminants, precise diagnostic criteria have been established [23,24,25]. Notably, non-dermatophytes have increasingly been recognized as significant contributors to the development of onychomycosis. The prevalence of non-dermatophyte infections is detailed in Section 3.7 below. Table 3 provides an overview of non-dermatophyte distribution across different countries.

### 2.3. Resistant Cutaneous Dermatophytosis

Over the past decade, there has been a noticeable increase in the prevalence of cutaneous dermatophytosis in India, with rates ranging from 6.0% to 61.5% depending on the specific region [2]. Notably, there is mounting evidence of the *T. mentagrophytes* complex replacing *T. rubrum* as the dominant species [26,27]. Of particular global concern is the emergence of *Trichophyton indotineae*, a newly identified species within the *T. mentagrophytes* complex (*T. mentagrophytes* ITS type VIII) [28]. A multicenter study conducted in India, involving clinically diagnosed dermatophytosis patients and molecular analysis of 351 specimens, reported that *T. indotineae* was the most prevalent species (90%), followed by *T. rubrum* (5%) and *T. mentagrophytes*/*T. interdigitale* (5%) [29]. *T. indotineae* has demonstrated reduced susceptibility to azole drugs and resistance to terbinafine, with over 50% of the strains exhibiting resistance [29,30]. Reports of *T. indotineae* have also emerged in the Middle East (Iran), Asia, and Europe in recent years [31]. Furthermore, other species within the *T. mentagrophytes* complex as well as *T. rubrum* have exhibited evidence of terbinafine-resistant and azole-resistant mechanisms attributed to SQLE gene mutations [32,33,34,35].

**Table 2:** Epidemiology of positive dermatophyte fungal isolations from cutaneous dermatophytosis in the 21st century.

Disease	Europe				South America	
	Switzerland [17] (2001–2018)	Ireland [18] (2001–2020)	Slovakia [36] (2014–2016)	Germany [37] (2014–2016)	Brazil [19] (2011–2019)	Argentina [38] (2002–2007)
	N = 10,958	N = 2263	N = 2103	N = 1252	N = 10,396	N = 1313
Tinea capitis	n = 830	n = 100	n = 44 (including tinea faciei)	n = 28	n = 435	n = 269
	1. *T. violaceum*	1. *T. tonsurans*	1. *T. mentagrophytes*	1. *T. mentagrophytes*	1. *T. tonsurans*	1. *M. canis*
	2. *M. audouinii*	2. *M. canis*	2. *M. canis*	2. *M. canis*	2. *M. canis*	2. *T. mentagrophytes*
	3. *T. soudanense*	3. *T. rubrum*	3. *M. audouinii*	3. *T. benhamiae*	3. *N. gypsea*	3. *N. gypsea*
Tinea faciei	n = 283	n = 10	-	n = 14	n = 151	-
	1. *T. mentagrophytes*	1. *T. tonsurans*		1. *T. rubrum*	1. *T. rubrum*	
	2. *T. benhamiae*	2. *T. verrucosum*		2. *T. benhamiae*	2. *N. gypsea*	
	3. *T. rubrum*	-		3. *M. canis*	3. *T. interdigitale*	
Tinea corporis	n = 1006	n = 64	n = 169	n = 185	n = 1148	n = 202
	1. *T. mentagrophytes*	1. *T. rubrum*	1. *T. tonsurans*	1. *T. rubrum*	1. *T. rubrum*	1. *T. rubrum*
	2. *T. rubrum*	2. *M. canis*	2. *T. rubrum*	2. *T. benhamiae*	2. *M. canis*	2. *T. mentagrophytes*
	3. *M. canis*	3. *T. tonsurans*	3. *T. mentagrophytes*	3. *T. interdigitale*	3. *T. tonsurans*	3. *M. canis*
Tinea manuum	n = 169	n = 19	n = 100	n = 48	n = 231	n = 26
	1. *T. rubrum*	1. *T. rubrum*	1. *T. rubrum*	1. *T. rubrum*	1. *T. rubrum*	1. *T. mentagrophytes*
	2. *T. mentagrophytes*	2. *T. verrucosum*	2. *T. mentagrophytes*	2. *T. interdigitale*	2. *T. interdigitale*	2. *T. rubrum*
	3. *T. benhamiae*	-	3. *T. tonsurans*	3. *T. benhamiae*	3. *T. tonsurans*	3. *Trichophyton* spp.
Tinea cruris	n = 427	n = 6	n = 245	-	n = 588	n = 53
	1. *T. rubrum*	1. *T. rubrum*	1. *T. rubrum*		1. *T. rubrum*	1. *T. rubrum*
	2. *T. mentagrophytes*	2. *E. floccosum*	2. *T. interdigitale*		2. *T. interdigitale*	2. *M. canis*
	3. *M. canis*	-	3. *E. floccosum*		3. *T. tonsurans*	3. *T. mentagrophytes*
Tinea pedis	n = 2439	n = 134	n = 649	n = 398	n = 3222	n = 77
	1. *T. rubrum*	1. *T. rubrum*	1. *T. rubrum*	1. *T. rubrum*	1. *T. rubrum*	1. *T. rubrum*
	2. *T. interdigitale*	2. *T. mentagrophytes*	2. *T. interdigitale*	2. *T. interdigitale*	2. *T. interdigitale*	2. *T. interdigitale*
	3. *E. floccosum*	3. *T. interdigitale*	3. *T. mentagrophytes*	3. *T. mentagrophytes*	3. *E. floccosum*	3. *Trichophyton* spp.
Onychomycosis	n = 5803	n = 1617	n = 896	n = 579	n = 4621	n = 671
	1. *T. rubrum*	1. *T. rubrum*	1. *T. rubrum*	1. *T. rubrum*	1. *T. rubrum*	1. *T. rubrum*
	2. *T. interdigitale*	2. *T. mentagrophytes*	2. *T. interdigitale*	2. *T. interdigitale*	2. *T. interdigitale*	2. *T. interdigitale*
	3. *T. soudanense*	3. *T. interdigitale*	3. *T. tonsurans*	-	3. *T. mentagrophytes*	3. *Trichophyton* spp.
**Diseases**	**Asia**					
	**Iran** [39] **(2008–2010)**	**Iran** [40] **(2010–2014)**	**India** [26] **(2014–2015)**	**China** [21] **(2004–2014)**	**Japan** [20] **(2016)**	**Thailand** [16] **(2014–2016)**
	**N = 777**	**N = 1535**	**N = 66**	**N = 588**	**N = 1268**	**N = 2350**
Tinea capitis	n = 15	n = 80	n = 5	n = 109	n = 15	n = 19
	1. *M. canis*	1. *T. tonsurans*	1. *T. tonsurans*	1. *M. canis*	1. *M. canis*	1. *T. rubrum*
	2. *T. tonsurans*	2. *T. mentagrophytes*	2. *T. violaceum*	2. *T. mentagrophytes*	2. *T. rubrum*	2. *T. mentagrophytes*
	3. *T. interdigitale*	3. *T. rubrum*	-	3. *T. violaceum*	3. *T. tonsurans*	3. *M. canis*
Tinea faciei	n = 9	-	-	n = 22	-	n = 50
	1. *T. tonsurans*			1. *T. mentagrophytes*		1. *T. rubrum*
	2. *M. canis*			2. *T. rubrum*		2. *T. mentagrophytes*
	3. *T. interdigitale*			3. *M. canis*		3. *M. canis*
Tinea corporis	n = 131	n = 242	n = 20	n = 61	n = 188	n = 276
	1. *T. interdigitale*	1. *T. tonsurans*	1. *T. interdigitale*	1. *T. rubrum*	1. *T. rubrum*	1. *T. rubrum*
	2. *T. rubrum*	2. *T. rubrum*	2. *T. tonsurans*	2. *M. canis*	2. *M. canis*	2. *M. canis*
	3. *M. canis*	3. *E. floccosum*	3. *M. gypseum*	3. *M. gypseum*	3. *T. interdigitale*	3. *T. mentagrophytes*
Tinea manuum	n = 16	n = 155	-	n = 20	n = 19	n = 54
	1. *T. interdigitale*	1. *T. tonsurans*		1. *T. rubrum*	1. *T. rubrum*	1. *T. rubrum*
	2. *T. rubrum*	2. *E. floccosum*		2. *M. canis*	2. *T. interdigitale*	2. *T. mentagrophytes*
	-	3. *T. verrucosum*		-	-	3. *M. canis*
Tinea cruris	n = 171	n = 457	n = 35	n = 72	n = 90	n = 198
	1. *E. floccosum*	1. *E. floccosum*	1. *T. interdigitale*	1. *T. rubrum*	1. *T. rubrum*	1. *T. rubrum*
	2. *T. rubrum*	2. *T. rubrum*	2. *T. tonsurans*	2. *M. gypseum*	2. *E. floccosum*	2. *T. mentagrophytes*
	3. *T. interdigitale*	3. *T. mentagrophytes*	3. *T. rubrum*	3. *T. mentagrophytes*	3. *N. gypsea*	3. *E. floccosum*
Tinea pedis	n = 353	n = 466	n = 4	n = 105	n = 665	n = 716
	1. *T. interdigitale*	1. *T. mentagrophytes*	1. *T. interdigitale*	1. *T. rubrum*	1. *T. rubrum*	1. *T. mentagrophytes*
	2. *T. rubrum*	2. *T. rubrum*	2. *T. tonsurans*	2. *T. mentagrophytes*	2. *T. interdigitale*	2. *T. rubrum*
	3. *E. floccosum*	3. *E. floccosum*	-	-	3. *E. floccosum*	3. *E. floccosum*
Onychomycosis	n = 82	n = 135	n = 2	n = 199	n = 290	n = 1137
	1. *T. rubrum*	1. *T. rubrum*	1. *T. interdigitale*	1. *T. rubrum*	1. *T. rubrum*	1. *T. rubrum*
	2. *T. interdigitale*	2. *T. mentagrophytes*	-	2. *T. mentagrophytes*	2. *T. interdigitale*	2. *T. mentagrophytes*
	3. *E. floccosum*	3. *E. floccosum*	-	-	3. *M. canis*	3. *T. tonsurans*

Note: Some studies identified *Trichophyton mentagrophytes* by conventional techniques whereas the others can distinguish *Trichophyton interdigitale* by molecular diagnosis.

**Table 3:** Prevalence of non-dermatophyte onychomycosis in various countries during the 21st century.

Organisms	Europe			South America		Africa		Asia			
	Switzerland [17] (2001–2018)	Greece [41,42] (2004–2015) (2015–2017)	Serbia [43] (2012–2014)	Guatemala [44] (2008–2011)	French Guiana [45] (2006–2009)	Ethiopia [46] (2015–2019)	Morocco [47] (2006–2010)	Iran [48] (2007–2014)	Israel [49] (2001–2015)	China [50] (2001–2020)	Thailand [51] (2014–2019)
	N = 17,175 *	N = 1450 **	N = 190 **	N = 4220 **	N = 205 *	N = 571 *	N = 1335 *	N = 648 *	N = 27,093 *	N = 32,190 *	N = 2740 **
*Acremonium* spp.	1078 ***	41	-	2	-	1	-	3	26	-	-
*Alternaria* spp.	-	-	-	1	-	3	-	3	15	-	-
*Aspergillus* spp.	-	1	2	11	2	28	14	108	53	958	-
*Cladosporium* spp.	-	-	-	3	-	14	-	2	1	-	-
*Fusarium* spp.	1078 ***	10	1	1	5	14	6	7	14	106	253
*Neoscytalidium dimidiatum*	-	-	2	-	29	11	27	-	-	-	360
*Penicillium* spp.	-	-	-	-	1	14	-	3	2	496	-
*Scopulariopsis brevicaulis*	-	48	2	8	2	4	-	8	22	45	-
Other molds	6996	-	1	6	5	-	20	9	24	522	-
Total	8074 (47%)	100 (6.9%)	8 (4.2%)	32 (0.8%)	44 (21.5%)	89 (15.6%)	67 (5%)	143 (22%)	157 (0.6%)	2127 (6.6%)	N/A

* Number of positive fungal isolations from onychomycosis without the mention of complete criteria for non-dermatophyte diagnosis. ** Number of patients diagnosed with onychomycosis with repetitive culture for non-dermatophyte infection. *** Number including both *Acremonium* spp. and *Fusarium* spp.

Although supporting evidence is limited, a previous study suggested that factors such as patient nonadherence, the widespread availability of topical antifungal-corticosteroid combinations, and improper use of antifungal agents may contribute to the promotion of antifungal resistance [52]. Another proposed mechanism of drug resistance in dermatophyte and non-dermatophyte infections is the formation of biofilms within the extracellular matrix. This phenomenon results in antimicrobial resistance, compromised function of host immune cells, and the formation of dermatophytomas on the nail plate [53].

## 3. Body Sites, Dermatophyte Species and Their Geographical Distribution

### 3.1. Tinea Capitis

Tinea capitis, a fungal infection of the scalp, is more commonly observed in school-age children than in adults. However, adult-onset tinea capitis has been associated with immunocompromised individuals [54]. This condition represents a major global public health concern, with varying prevalence rates across countries. The frequency of the disease ranges from 0.4% to 87.7% in Africa, 0.2% to 74.0% in America, 0.04% to 78.6% in Europe, and 0.01% to 91.2% in Asia [55].

In the African continent, tinea capitis exhibits regional variations in the predominant pathogens. In the northern region, *T. violaceum* and *M. canis* have been identified as the primary pathogens [56]. In the eastern and southern regions, *T. violaceum* and *M. audouinii* prevail, while in the western region, *Trichophyton soudanense* and *M. audouinii* are commonly observed [3].

In the Americas, *Trichophyton tonsurans* is the predominant causative organism in the United States, while *M. canis* is more prevalent in Mexico and Central America. These two organisms also dominate in South America [55]. European countries exhibit a predominance of zoophilic dermatophytosis, with *M. canis* and *T. mentagrophytes* being common across regions. *T. soudanense* and *T. tonsurans* are significant agents in France and Ireland, respectively, while *T. violaceum* is reported as the most prevalent species in Switzerland, Scotland, and Sweden [55].

Across Asia, there is considerable variation in the main pathogens. *T. violaceum* and *T. tonsurans* predominate in India, while *T. mentagrophytes*, *T. violaceum*, *T. verrucosum*, and *M. canis* have been identified as the primary agents in Western Asia. Eastern Asia shows a predominance of *M. canis* and *T. violaceum*, while Southeast Asia is characterized by *M. canis* as the chief pathogen. Notably, Thailand differs from other Southeast Asian countries, with *T. rubrum* and *T. mentagrophytes* being the predominant organisms [16,55,57].

### 3.2. Tinea Faciei

The prevalence and causative agents of tinea faciei vary across regions. The reported isolation rates range from 0.4% to 4.2% in various parts of the world [16,17,18,19,20,21,26,36,37,38,39,40,58,59], with more than 50% of cases observed in patients younger than 12 years of age [37,60]. In Europe, a study conducted in Portugal identified *M. audouinii*, *T. soudanense*, and *T. rubrum* as the main pathogenic agents [60]. Other European countries have reported zoophilic dermatophytes as the primary causative agents, such as *T. mentagrophytes* in Switzerland and Slovakia, and *M. canis* in Italy and Greece [17,36,61,62]. *T. rubrum* was predominant in Germany and Brazil [19,37]. In Asia, *Trichophyton* spp., including *T. rubrum*, *T. mentagrophytes*, and *T. tonsurans*, were identified as the main pathogenic agents [16,21,39,63].

Tinea barbae, a relatively rare form of tinea faciei, refers to a fungal infection affecting the hair follicles in the beard area of adult men. The main causative agents identified include *Trichophyton* spp., such as *T. mentagrophytes*, *T. rubrum*, *T. tonsurans*, and *Trichophyton benhamiae* [64,65,66,67]. Cases have been reported in Europe, the Americas, and Asia.

### 3.3. Tinea Corporis

Tinea corporis, a fungal infection of the body, poses a significant global public health challenge. The primary causative agents of tinea corporis worldwide in recent years are *Trichophyton* spp., with *T. rubrum* being the most common, followed by the *T. mentagrophytes* complex, *M. canis*, *T. tonsurans*, and *T. benhamiae* [16,17,18,19,20,21,37,39]. In Africa, the estimated prevalence ranges from 2% to 41% [3]. In other regions, the prevalence of tinea corporis among isolated dermatophytes is estimated to be between 2.8% and 30.3% (Table 2) [16,17,18,19,20,21,26,36,37,38,39,40,58,59]. Notably, in India, there has been a shift in prevalence over the past two decades, with *T. mentagrophytes* emerging as a replacement for *T. rubrum* [27,68].

Tinea corporis manifests in various clinical variants. Majocchi granuloma, which primarily affects females in a ratio of 3:1 and occurs in the age range of 20–35 years, represents a deep dermatophyte folliculitis. Trauma (such as leg shaving) and immunosuppression are associated factors. While *T. rubrum* is the usual causative agent, other species of *Trichophyton* spp. or *Microsporum* spp. have been reported [69].

Tinea gladiatorum is a dermatophytosis commonly observed in contact sports athletes, wrestlers, and their family members. The prevalence of dermatophytosis in wrestlers varies from 2.4% to 100%, as reported in the United States, Iran, and Japan, with over 90% of cases being asymptomatic carriers [70,71]. The primary pathogenic agent in tinea gladiatorum is *T. tonsurans*. Factors associated with this condition include excessive sweating, poor hygiene, and contaminated training mats [70].

Tinea imbricata presents a unique clinical appearance characterized by concentric annular rings with flaking skin. It is caused by *Trichophyton concentricum* and has been reported in Asia, Oceania, the Middle East, and South America [72]. The prevalence of tinea imbricata varies across countries and regions. The highest prevalence (18.3–20.1%) has been documented among indigenous people or tribes in Malaysia and Indonesia [73,74].

### 3.4. Tinea Cruris

Tinea cruris, a common fungal infection affecting the groin region, is prevalent worldwide, particularly in warm and humid areas [58]. The estimated prevalence of cutaneous fungal infections ranges from 0.3% to 53.0% [16,17,18,19,20,21,26,36,37,38,39,40,58,59]. High prevalence rates exceeding 20% have been documented in regions such as Asia and the Middle East, including countries such as China and Iran [39,58]. In India, tinea cruris has been reported as the most prevalent form of chronic and recurrent dermatophytosis, affecting up to 80% of cases [75].

The primary causative agent worldwide is *T. rubrum*, followed by other common pathogens such as *T. mentagrophytes* and *E. floccosum*. Interestingly, the epidemiology of tinea cruris exhibits regional variations. Evidence suggests a decline in the prevalence of *E. floccosum* in certain regions, such as Germany, Chile, and Thailand, where the prevalence has decreased from 12.6% to 2.7% over 30 years [8,11,16,76]. However, in Iran, *E. floccosum* continues to be the chief pathogen responsible for tinea cruris [39,40]. Major predisposing factors include excessive sweating, diabetes, and obesity [77]. The development of the disease can be influenced by autoinfection from tinea pedis and onychomycosis, as these conditions share similar causative agents.

### 3.5. Tinea Manuum

Tinea manuum is a cutaneous fungal infection affecting the hand. The estimated prevalence of cutaneous dermatophytosis is 0.8% to 12.6% [16,17,18,19,20,21,26,36,37,38,39,40,58,59]. The most common pathogen worldwide is *T. rubrum*, followed by zoophilic dermatophytes such as *T. mentagrophytes* and *M. canis*. In recent years, there have been emerging case reports of *Trichophyton erinacei* from various regions across the globe [78,79,80,81]. This particular agent is frequently isolated from hedgehogs and can cause widespread infection with increased virulence among immunocompromised patients [82].

### 3.6. Tinea Pedis

Tinea pedis, commonly known as fungal foot infection, typically affects the feet, primarily involving the sole, interdigital toe web, and dorsal surface of the foot. Major risk factors for this condition include older age, male sex, obesity, low level of education, low income, physical disability, and specific populations exposed to sweating, occlusive footwear, or contaminated floors in communal settings [83,84,85,86]. The estimated prevalence of tinea pedis among cutaneous dermatophytosis ranges from 5.9% to 52.4% [16,17,18,19,20,21,26,36,37,38,39,40,58]. Dermatophytes, particularly *Trichophyton* species, account for the majority of causative agents, comprising approximately 36.8% to 70.5% [87,88,89]. *T. rubrum* is the most common pathogen worldwide, followed by *T. mentagrophytes*/*T. interdigitale*. The prevalence of these *T. mentagrophytes* complex has shown an increasing trend in the past 20 years [85]. Previous studies in specific populations, such as naval cadets, have reported *T. mentagrophytes*/*T. interdigitale* as the most prevalent species [86,90]. Non-dermatophytes have been reported in 8.02% to 57.9% of cases, with higher rates observed in tropical regions such as Africa and Southeast Asia [87,88,89]. Common agents causing non-dermatophyte infections on the feet include *Neoscytalidium dimidiatum* and *Fusarium* species. Tinea pedis is also frequently observed as a concomitant infection with onychomycosis [85,91].

### 3.7. Onychomycosis

Onychomycosis is a chronic or relapsing fungal infection of the nail, predominantly affecting geriatrics. The global prevalence in the general population is 5.5%, varying depending on population heterogeneity [92]. In the United States, the estimated prevalence ranges from 1.6% to 13.8% [93,94]. A systematic review reported a mean prevalence of 4.3% based on population-based studies in Europe and North America, while hospital-based studies showed a mean prevalence of 8.9% [95]. Recent studies on cutaneous fungal infections have identified onychomycosis as having the highest prevalence among other cutaneous dermatophytosis, ranging from 3.0% to 82.9% [16,17,18,19,20,21,26,36,37,38,39,40,58].

The dermatophyte group is the most commonly isolated agent worldwide and is responsible for 60% to 70% of cases. *T. rubrum* is the predominant species (accounting for approximately 50% or more), followed by *T. mentagrophytes* and *T. interdigitale* [95,96]. Although *M. canis* is a rare etiological agent, it has been reported in cases of fingernail onychomycosis, particularly in younger patients with a history of contact with an infected pet [97]. Non-dermatophyte onychomycosis is more challenging to treat than dermatophyte infections due to the high failure rate of antifungal therapy [51,53].

Non-dermatophytes are globally recognized as causative agents, with their prevalence ranging from 0.8% to 65.8% [44,95,98,99,100]. In specific regions, such as Brazil [99], Sri Lanka [101], and Thailand [88], non-dermatophytes have been detected in up to 51.6% to 68.2% of cases. In contrast, Europe reports a lower prevalence, ranging from 4% to 7% [41,42,43]. The spectrum of pathogenic agents also varies across regions. In Europe, *Scopulariopsis brevicaulis*, *Aspergillus* spp., *Acremonium* spp., and *Fusarium* spp. are the most commonly identified non-dermatophytes [41,42,43,102], with *Fusarium* spp. accounting for a prevalence of up to 7.5% to 9.2% of onychomycosis cases [51,102,103,104]. In tropical regions such as Thailand, *N. dimidiatum* is the primary pathogen [88,98], reported with a prevalence of 13% in onychomycosis cases [51]. Table 3 provides an overview of other pathogenic non-dermatophytes observed across countries.

Risk factors associated with onychomycosis include the following [92]:

Patients’ medical attributes: advanced age, genetic susceptibility, foot deformities, and comorbidities such as diabetes, immunosuppression, venous insufficiency, peripheral arterial diseases, malignancy, and obesity.

Dermatologic conditions: previous or concurrent tinea pedis, psoriasis, and hyperhidrosis.

Exogenous factors: trauma, poor nail grooming, participation in sports activities, occupational exposure, smoking, and wearing occlusive footwear.

A previous study on diabetes patients found that older age, agricultural-related activities, family history of dermatophytosis, and comorbidities such as coronary heart disease were associated with onychomycosis and tinea pedis [90].

## 4. Clinical Presentations

### 4.1. Tinea Capitis

Tinea capitis presents various clinical manifestations depending on the causative agents and the severity of the disease. It predominantly affects children aged 6 months to 12 years [105]. The clinical variants are categorized into three forms based on the level of hair shaft invasion, and in outbreak infections, a mixed-type clinical pattern may be observed [106]. In adult patients, nearly 40% exhibit concurrent dermatophytosis at other sites [57].

**Figure 1:**
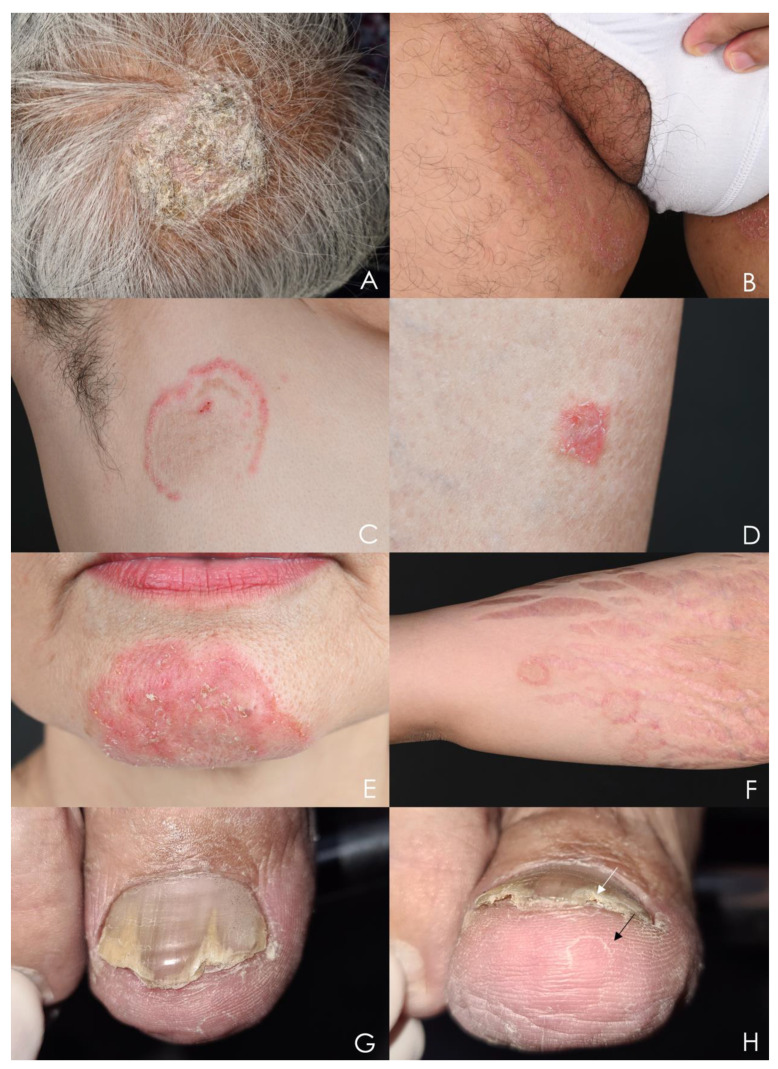
Clinical presentations of cutaneous fungal infection. (**A**) Tinea capitis: Gray patch on the vertex of the scalp. (**B**) Tinea cruris: Multiple annular scaly erythematous patches on the groin. (**C**) Tinea incognito: Ring-within-a-ring appearance. (**D**) Tinea corporis: Annular scaly erythematous macule with inflammation on the right leg caused by *Microsporum canis*. (**E**) Tinea faciei: Multiple scaly erythematous concentric rings on the chin caused by *Microsporum canis*. (**F**) Tinea corporis: Multiple red-rubber-ring macules on the right arm caused by *Trichophyton mentagrophytes*. (**G**) Dorsal view of the right big toenail: Onychomycosis caused by *Trichophyton mentagrophytes*, showing dermatophytoma and longitudinal striae adjacent to the dermatophytoma. (**H**) Hyponychium view of the right big toenail of the same patient: Sulfur-nugget-like subungual debris (white arrow) concurrent with tinea pedis presenting as an annular scaly macule (black arrow).

The ectothrix form is caused by fungal pathogens that invade the mid-follicle level of the hair shaft and form a sheath around the hair [105]. Clinical presentations include solitary or multiple gray patches with scales and circular alopecia patches with breaking hair shafts above the scalp level (Figure 1A). The causative agents are *Microsporum* spp. and certain *Trichophyton* spp. In *M. audouinii* infection, the degree of inflammation is minimal, resulting in only fine scales. Zoophilic organisms such as *M. canis* or *Trichophyton verrucosum* commonly lead to greater inflammation and can present with pustules, furuncles, or kerion. Kerion is a severe, painful inflammatory mass that can cause scarring alopecia.

The endothrix form typically occurs when hyphae invade the hair shaft without damaging the cortex and cuticle [105]. This form is characterized by multiple alopecia patches with broken hairs at the level of the hair follicle opening, resulting in the appearance of black dots. Common causes include *T. tonsurans*, *T. soudanense*, and *T. violaceum*.

Favus form is a fungal infection caused by *T. schoenleinii*. It is characterized by fungal hyphae growing longitudinally along the hair shaft, with air spaces within [105,107]. Favus presents with scutula, which are yellow cup-shaped crusts containing the hyphae, on the follicular opening of the hair shaft. The lesions can sometimes enlarge, forming a confluent mass of pale powdery crusts. Without treatment, chronic disease progression can lead to scarring alopecia.

### 4.2. Tinea Coporis, Tinea Faciei, and Tinea Cruris

The clinical presentation of superficial fungal infections of the skin is characteristic and typical. Tinea corporis, tinea faciei, and tinea cruris typically manifest as solitary or multiple annular scaly erythematous macules and patches that progress with central clearing (Figure 1B). An additional sign of anthropophilic cutaneous dermatophytosis is a ring-within-a-ring appearance (Figure 1C). In contrast, zoophilic dermatophytosis exhibits more inflammation and rapid progression (Figure 1D,E). Clues that may indicate zoophilic dermatophytosis include a history of contact with pets, involvement of exposed areas, presence of vesicles or pustular lesions, and a red-rubber-ring appearance (Figure 1F) [108].

Other forms of tinea corporis present with distinct clinical manifestations. Majocchi granulomas appear as erythematous or violaceous papules, plaques, or nodules, typically on the legs and forearms. These lesions are often painful or pruritic and exhibit more inflammation despite primarily being caused by anthropophilic dermatophytes [69]. Tinea imbricata starts as a solitary concentric, ring-like scaly macule with slow progression into a patch or plaque with or without pruritus. The rash is commonly found on the trunk or extremities [72]. Patients who have previously applied topical corticosteroids may develop a condition known as tinea incognito. Using steroids can modify the rash, resulting in the disappearance of an annular scaly border and the progression to a diffuse erythematous rash or follicular papules or pustules that mimic other dermatologic conditions [109]. Although approximately half of the patients may present with a ring-within-a-ring appearance, the difference is insignificant compared to patients without prior topical steroid usage [108].

### 4.3. Tinea Manuum, Tinea Pedis

Dermatophytosis on the hands and feet exhibits distinct clinical features depending on the sites of infection. The symptoms and signs of dorsal surface infection are similar to those observed in tinea corporis due to the similarities in skin structure [110]. However, the thick keratinized epidermis and numerous eccrine glands on the palms and soles give rise to different manifestations [107].

Tinea manuum commonly presents as dry, scaly macules or patches, sometimes accentuated in the flexural creases of the palms. These lesions can be localized or diffuse and may affect one or both hands. In cases of zoophilic dermatophytosis infection, more inflammatory lesions, such as erythematous, pustular, or vesicular formations, may occur. Tinea manuum can also manifest as two feet-one hand syndrome, indicating concurrent tinea pedis and onychomycosis [111].

Tinea pedis, or athlete’s foot, exhibits three clinical subtypes and can involve one or both feet. The moccasin type is a chronic mild form characterized by small collarette scaly macules or diffuse, dry, scaly lesions with or without inflammation. This type is commonly associated with anthropophilic dermatophytosis, particularly *T. rubrum* infection. The vesicular type resembles a dyshidrotic reaction and spontaneously regresses with collarette scaling. In more severe cases, inflammatory bullous lesions may develop. This more inflammatory type is usually caused by zoophilic dermatophytosis. The last clinical subtype is interdigital tinea pedis, which presents as scaly whitish patches with fissuring or moist maceration in the interdigital toe web spaces. The fourth interdigital web space is commonly affected. Concurrent onychomycosis, especially at the same site affected by tinea pedis, is common, highlighting the need for toenail examination in most cases [107].

### 4.4. Onychomycosis

Onychomycosis primarily affects the toenails in geriatrics, with the great toenail being the most commonly involved [92]. However, fingernail onychomycosis has a higher prevalence among younger individuals [112]. Distal lateral subungual onychomycosis is the most frequently observed clinical subtype, followed by total nail dystrophy, proximal subungual onychomycosis, and superficial white onychomycosis [50]. Typical presentations are onycholysis, subungual hyperkeratosis, and nail discoloration [92]. Chronic and long-standing onychomycosis can lead to extensive onychodystrophy.

In distal lateral subungual onychomycosis, a characteristic feature called dermatophytoma can be observed. It presents as a fungal abscess, appearing as a white- or yellow-colored, triangular, longitudinal streak in the nail plate (Figure 1G). However, dermatophytosis-like traumatic onychodystrophy can also occur without fungal infection. Differentiation between dermatophytoma onychomycosis and other conditions can be guided by specific characteristics. Dermatophytoma onychomycosis typically exhibits inhomogeneous colored, longitudinal striae adjacent to the dermatophytoma, as well as sulfur-nugget-like subungual debris visible on hyponychium view (Figure 1H) [113]. Approximately one-third of patients with onychomycosis also have concomitant tinea pedis (Figure 1H) [85]. Therefore, considering the presence of tinea pedis can assist in the clinical diagnosis, and simultaneous treatment of both conditions is necessary for complete resolution.

One of the challenges in managing onychomycosis lies in distinguishing dermatophyte from non-dermatophyte infections through physical examination alone. Unfortunately, there are no significant differences in clinical nail characteristics. However, it is worth noting that patients with non-dermatophyte onychomycosis typically lack fungal glabrous skin infections in areas other than the feet [98]. For instance, compared to *Neoscytalidium* onychomycosis, *Fusarium* onychomycosis is more commonly associated with a history of pedicure, predominant lateral nail involvement, and the absence of concurrent foot infection [51].

## 5. Diagnostic Testing

### 5.1. Direct Microscopic Examination

**Figure 2:**
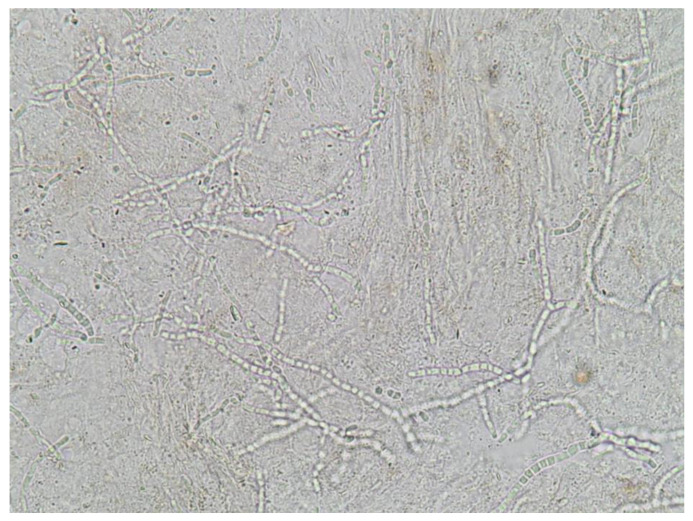
Potassium hydroxide examination with the 40× objective revealing branching septate hyphae under a light microscope.

A mycological examination is essential for a definitive diagnosis prior to initiating treatment. This method has proven cost-effective, considering the potential risks associated with systemic drug use [114]. Potassium hydroxide (KOH) examination is a simple bedside technique that involves collecting a sample by scraping the keratin from an active border area of the rash or nail. The sample is then prepared with KOH and examined under a light microscope. Branching septate hyphae can be observed on the slide (Figure 2). Although the accuracy of this method relies on the experience of technicians [115], conducting repeated examinations can improve diagnostic potential [116]. The sensitivity ranges from 67% to 93%, while the specificity ranges from 38% to 78% [92].

### 5.2. Histopathology

**Figure 3:**
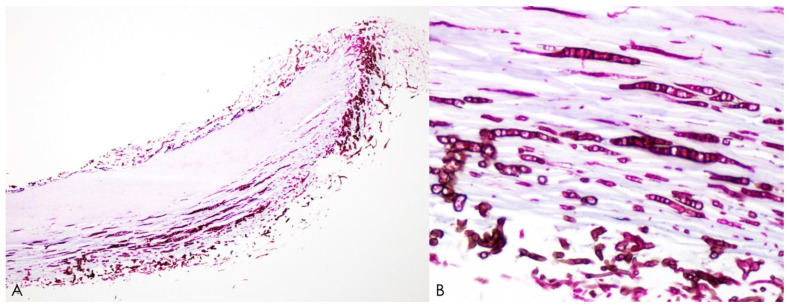
Histopathological analysis of nail clippings from *Neoscytalidium dimidiatum*-induced onychomycosis using periodic acid–Schiff staining. Microscopic examination at low power with the 4× objective (**A**) and high power with the 40× objective (**B**) revealing black-brown fungal hyphae invading the nail plate.

Histopathology with Periodic acid-Schiff or Grocott methenamine silver stains obtained from nail clipping is the convenient method, and high sensitivity is reported (92–98.8%) [43]. The fungal hyphae penetrating into the nail plate is obvious, allowing for diagnosis (Figure 3). However, both KOH examination and histology cannot identify whether the fungus is viable or not.

### 5.3. Fungal Culture

Fungal culture is a definitive method for identifying fungi and detecting viable organisms. Sabouraud dextrose agar is commonly used as a culture medium for non-dermatophyte growth, while Sabouraud dextrose agar containing cycloheximide is used for dermatophyte growth [92]. This technique is widely available in dermatology clinics worldwide. The fungal species can be identified by observing the distinct morphological colonies on the culture media and examining the characteristics of conidia under a light microscope. However, there are limitations: the time required for fungal identification from colony cultures, false negative results, and the potential growth of various non-dermatophyte contaminants, especially in nail samples [117]. The overall sensitivity ranges from 23.8% to 79.3%, while the specificity ranges from 83% to 100% [92,118].

For the diagnosis of non-dermatophyte onychomycosis, it has been proposed that at least three of the following six criteria be met [102]:

- identification of fungal hyphae in KOH examination
- isolation of non-dermatophytes in culture
- repeated isolation of the same non-dermatophytes in fungal culture
- growth of the inoculum in at least 5 out of 20 fragments
- absence of dermatophyte isolation in culture
- supporting evidence of onychomycosis from histology

While these criteria are widely used for diagnosis, a previous study reported that mixed dermatophyte and non-dermatophyte infections are common, with a prevalence ranging from 20% to 40% in onychomycosis [119]. The most common mixed organisms were *T. mentagrophytes* and *N. dimidiatum* (53.2%), followed by *T. rubrum* and *N. dimidiatum* (21.3%). Treatment for mixed infections usually requires a longer duration than pure dermatophyte infections.

### 5.4. Molecular Diagnosis

Molecular diagnosis has become a well-known technique in scientific research, aiming to enhance the speed, cost-effectiveness, and convenience of identifying fungi with minimal technical effort [120]. Various molecular techniques have been developed: conventional polymerase chain reaction (PCR), real-time PCR, nested PCR, multiplex PCR, PCR enzyme-linked immunosorbent assay, and PCR-restricted fragment length polymorphism. Notably, significant advancements have been made in molecular characterizations, such as DNA extraction and DNA sequencing, utilizing internal transcribed spacer polymorphisms within the ITS rDNA regions. These advancements have enabled reliable differentiation of various species, particularly in cases of *T. rubrum* and other dermatophyte infections [121,122]. These methods offer high sensitivity (95%) and specificity (100%) for dermatophyte species identification [92]. Furthermore, advances have been made in identifying non-dermatophyte pathogens [35,120]. The application of multiplex PCR for the diagnosis of *Fusarium* spp. onychomycosis has demonstrated high efficiency, sensitivity, and specificity [123]. However, challenges remain, including the requirement for specialized laboratory equipment and the difficulty in differentiating true pathogens from contaminants [102]. Nevertheless, commercial PCR kits have shown promise through their cost-effectiveness and availability, with increasing usage in several countries [92].

### 5.5. Dermoscopy

Dermoscopy is a valuable tool for dermatologic examinations at the bedside, aiding the diagnosis of onychomycosis and tinea capitis. Dermoscopy findings also help diagnose tinea capitis and distinguish it from other types of alopecia [113]. The hyponychium view reveals subungual hyperkeratosis with sulfur nuggets, characterized by yellow clumping debris with a crumbled appearance, supporting the diagnosis of onychomycosis [124]. On the dorsal view, distinct features such as distal subungual longitudinal streaks, longitudinal striae adjacent to dermatophytoma, spikes at the proximal margin of an onycholytic area, and brown and black nail discoloration are indicative of onychomycosis [124,125].

Dermoscopy findings also help diagnose tinea capitis and distinguish it from other types of alopecia. Multiple comma-shaped hairs, dystrophic and elbow-shaped hairs, varying levels of broken hair resembling Morse code, and other specific hair patterns strongly support the diagnosis of tinea capitis [105].

### 5.6. Wood’s Light Examination

**Figure 4:**
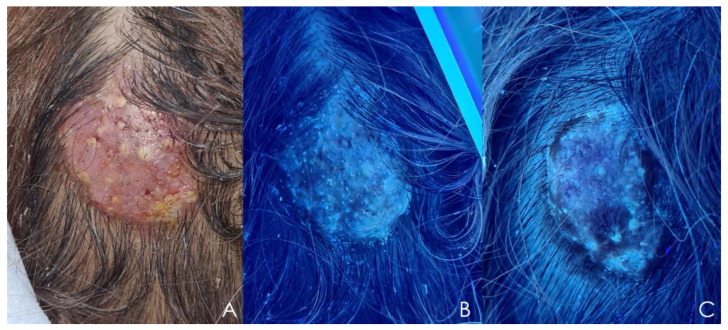
Kerion caused by *Microsporum canis*. (**A**) Clinical presentation: Solitary inflammatory mass with alopecia. (**B**) Wood’s light examination of the same patient: Blue-greenish fluorescence. (**C**) Wood’s light examination after 2 weeks of fluconazole treatment: Disappearance of fluorescence.

Wood’s light examination can support the diagnosis of cutaneous dermatophytosis, particularly tinea capitis caused by *Microsporum* spp. and *T. schoenleinii* infections [105]. *Microsporum* spp. emit a blue-green fluorescence (Figure 4B), while *T. schoenleinii* exhibits a light-blue fluorescence under Wood’s light [126].

## 6. Conclusions

Cutaneous fungal infections from both dermatophyte and non-dermatophyte pathogens pose a significant global public health concern. The epidemiology of these infections is dynamic, with an increasing prevalence of non-dermatophyte infections and the emergence of resistant strains of dermatophytosis. This review provides an updated overview of the epidemiology, clinical manifestations, and diagnostic methods, offering valuable insights for an accurate diagnosis before initiating treatment. Achieving a precise diagnosis is crucial for effective management and can contribute to the prevention of antifungal resistance. Further research on resistant superficial fungal infections is imperative to enhance global prevention strategies and address this evolving healthcare challenge.

## Figures and Tables

**Table 1:** Dermatophytes and the habitat according to phylogenetic tree using the ITS rDNA region [5,6].

Habitat	Anthropophilic Dermatophytes	Zoophilic Dermatophytes	Geophilic Dermatophytes
Dermatophyte species	*Trichophyton concentricum*	*Trichophyton benhamiae*	*Nannizzia aenygmaticum*
	*Trichophyton indotineae*	*Trichophyton bullosum*	*Nannizzia corniculata*
	*Trichophyton interdigitale*	*Trichophyton equinum*	*Nannizzia fulva*
	*Trichophyton rubrum*	*Trichophyton eriotrephon*	*Nannizzia gypsea*
	*Trichophyton schoenleinii*	*Trichophyton erinacei*	*Nannizzia incurvata*
	*Trichophyton soudanense*	*Trichophyton mentagrophytes*	*Nannizzia praecox*
	*Trichophyton tonsurans*	*Trichophyton quinckeanum*	*Paraphyton cookei*
	*Trichophyton violaceum*	*Trichophyton simii*	*Paraphyton cookiellum*
	*Epidermophyton floccosum*	*Trichophyton verrucosum*	*Arthroderma ciferrii*
	*Microsporum andouinii*	*Nannizzia nana*	*Arthroderma cuniculi*
	*Microsporum ferrugineum*	*Nannizzia persicolor*	*Arthroderma curreyi*
	*Arthroderma onychocola*	*Paraphyton mirabile*	*Arthroderma eboreum*
		*Lophophyton gallinae*	*Arthroderma gertleri*
		*Microsporum canis*	*Arthroderma gloriae*
		*Arthroderma amazonicum*	*Arthroderma insingulare*
		*Arthroderma flavescens*	*Arthroderma lenticulare*
		*Arthroderma redellii*	*Arthroderma melis*
		*Arthroderma vespertilii*	*Arthroderma multifidum*
			*Arthroderma phaseoliforme*
			*Arthroderma quadrifidum*
			*Arthroderma thuringiensis*
			*Arthroderma tuberculatum*
			*Arthroderma uncinatum*

## Data Availability

No new data were created or analyzed in this study. Data sharing does not applicable to this article.
